# Multi-Agent Reinforcement Learning in Games: Research and Applications

**DOI:** 10.3390/biomimetics10060375

**Published:** 2025-06-06

**Authors:** Haiyang Li, Ping Yang, Weidong Liu, Shaoqiang Yan, Xinyi Zhang, Donglin Zhu

**Affiliations:** 1 High-Tech Institute of Xi’an, Xi’an 710038, China; li_haiyang77@163.com (H.L.); yyp_ing@163.com (P.Y.); 13333775025@163.com (W.L.); yanshaoqiang668@163.com (S.Y.); 15542792723@163.com (X.Z.); 2 School of Computer Science and Technology, Zhejiang Normal University, Jinhua 321004, China

**Keywords:** multi-agent reinforcement learning, game theory, evolutionary computation, stochastic games

## Abstract

Biological systems, ranging from ant colonies to neural ecosystems, exhibit remarkable self-organizing intelligence. Inspired by these phenomena, this study investigates how bio-inspired computing principles can bridge game-theoretic rationality and multi-agent adaptability. This study systematically reviews the convergence of multi-agent reinforcement learning (MARL) and game theory, elucidating the innovative potential of this integrated paradigm for collective intelligent decision-making in dynamic open environments. Building upon stochastic game and extensive-form game-theoretic frameworks, we establish a methodological taxonomy across three dimensions: value function optimization, policy gradient learning, and online search planning, thereby clarifying the evolutionary logic and innovation trajectories of algorithmic advancements. Focusing on complex smart city scenarios—including intelligent transportation coordination and UAV swarm scheduling—we identify technical breakthroughs in MARL applications for policy space modeling and distributed decision optimization. By incorporating bio-inspired optimization approaches, the investigation particularly highlights evolutionary computation mechanisms for dynamic strategy generation in search planning, alongside population-based learning paradigms for enhancing exploration efficiency in policy refinement. The findings reveal core principles governing how groups make optimal choices in complex environments while mapping the technological development pathways created by blending cross-disciplinary methods to enhance multi-agent systems.

## 1. Introduction

**Figure 1:**
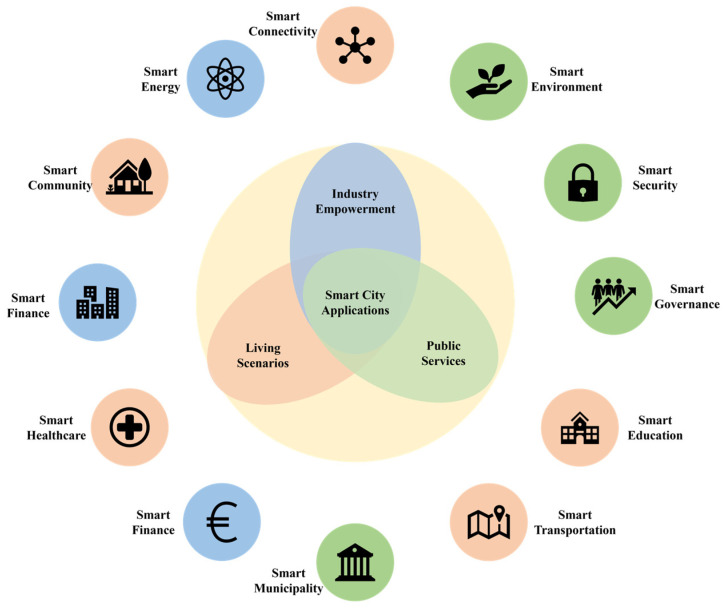
Smart city application scenarios.

The evolutionary trajectory of artificial intelligence has transitioned from symbolic reasoning foundations through statistical learning paradigms, ultimately achieving transformative progress in single-agent decision-making within constrained environments via deep learning breakthroughs. When operating in open dynamic environments that typify real-world applications, intelligent systems exhibit substantially heightened complexity in multi-agent collaborative decision-making processes. The smart city deployment architecture illustrated in Figure 1 encapsulates three critical operational challenges: autonomous vehicle right-of-way optimization [1,2], UAV swarm collision avoidance protocols [3], and intelligent grid demand-response coordination [4,5]. These interconnected scenarios fundamentally require emergent strategic co-evolution mechanisms among cyber-physical agents operating under shared environmental constraints. These systems face fundamental theoretical limitations as conventional reinforcement learning frameworks developed for single-agent contexts struggle to address the tripartite challenges of environmental state dynamism, strategic space dimensional escalation, and equilibrium configuration heterogeneity arising from emergent collective intelligence in multi-agent ecosystems [6,7].

The extension of traditional reinforcement learning to multi-agent domains confronts fundamental theoretical limitations. The primary challenge stems from policy interdependencies among agents inducing continuous dynamic shifts in environmental state transitions and reward mechanisms, thereby invalidating the Markov assumption. This complexity manifests when transportation systems experience global congestion pattern reconstruction through nonlinear interactions of localized routing decisions made by individual vehicles responding to real-time conditions. A secondary barrier emerges from the dimensional catastrophe inherent in agent population growth, where exponential expansion of strategy spaces overwhelms conventional Q-learning algorithms’ exploration capacity despite contemporary computational resources. The most profound impediment resides in equilibrium polymorphism—the coexistence of Nash equilibria, correlated equilibria, and diverse solution concepts that engender theoretical ambiguities in convergence guarantees and equilibrium selection criteria. These multifaceted challenges collectively underscore a paradigm-shifting imperative: multi-agent decision optimization necessitates transcending single-agent cognitive frameworks.

Addressing these complexities requires synergistic methodological integration. Game theory furnishes rigorous mathematical formalisms for strategic interactions through its equilibrium analysis framework, enabling precise characterization of competitive–cooperative dynamics. Reinforcement learning contributes data-driven optimization mechanisms for navigating high-dimensional continuous decision spaces via trial-and-error paradigms. Biological collective intelligence principles—exemplified by ant colony foraging optimization and avian flocking collision avoidance—offer bio-inspired strategies for resolving exploration–exploitation tradeoffs. These tripartite components form an interdependent helix architecture comprising formal interaction modeling through game-theoretic constructs, strategic space optimization via reinforcement learning mechanisms, and adaptive exploration inspired by swarm intelligence principles. This integrated approach facilitates balanced advancement in agent system design, harmonizing theoretical interpretability with computational tractability.

Recent theoretical advancements in stochastic games and their extended formulations have established a unified analytical framework for dynamic environment modeling [8]. Concurrently, the integration of deep neural architectures with curriculum learning mechanisms has substantially enhanced strategy representation efficiency and training robustness [9]. Furthermore, synergistic innovations bridging evolutionary computation and game-theoretic equilibrium analysis have established novel pathways for strategic convergence assurance in high-dimensional spaces. While these developments have partially mitigated decision-making challenges in multi-agent systems, a persistent disconnect persists between the compartmentalization of theoretical frameworks and the escalating complexity of real-world applications [10,11]. This study conducts a systematic examination of multi-agent reinforcement learning’s evolutionary trajectory through tripartite analytical lenses—value function decomposition, policy space architecture, and search mechanism optimization—while elucidating how the synergistic integration of game-theoretic principles and biological intelligence paradigms drives collaborative decision-making innovation. These insights ultimately furnish theoretical foundations and methodological frameworks for developing complex intelligent systems, ranging from urban cognitive infrastructures to industrial cyber-physical ecosystems.

This study is architecturally organized as follows: Chapter 2 elucidates the foundational theoretical framework integrating game theory with MARL. Chapter 3 conducts a systematic deconstruction of classical reinforcement learning algorithms and the evolution of cutting-edge methodologies through tripartite analytical dimensions—value function approximation, policy optimization, and search-based decision-making—with particular emphasis on delineating how collective intelligence principles drive algorithmic innovation. Chapter 4 critically examines implementation challenges in MARL applications, while Chapter 5 provides conclusive synthesis and prospective research directions.

## 2. Theoretical Foundation of MARL

### 2.1. Single-Agent Reinforcement Learning

Reinforcement learning (RL) constitutes a computational paradigm where an autonomous agent learns optimal behavioral policies through experiential exploration in environmental interactions. This framework operates through iterative cycles: The agent selects actions based on environmental states, subsequently triggering state transitions that generate reward signals. These feedback mechanisms enable the agent to progressively optimize its behavioral policy through successive environmental engagements, ultimately achieving either cumulative reward maximization or specified operational objectives. The fundamental learning mechanism resides in the agent’s capacity to balance environmental exploration with policy exploitation during this interactive process.

**Figure 2:**
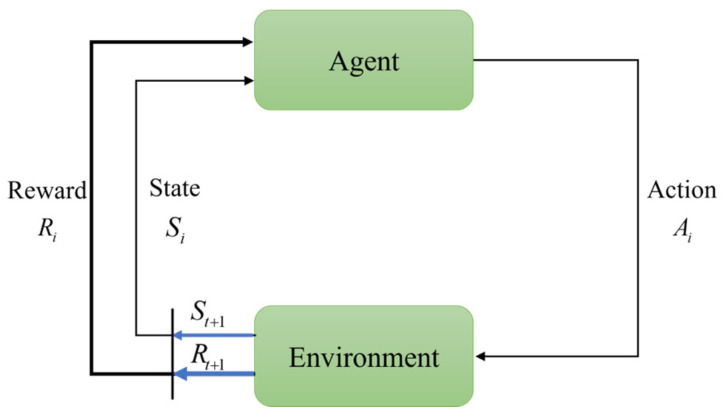
Modeling Markov Decision Process interactions between RL agents and the environment.

As illustrated in Figure 2, the agent–environment interaction paradigm is formally represented as a Markov Decision Process (MDP), mathematically defined by the quadruple $\langle S,A,P,R\rangle$. The fundamental components comprise the following:

*S*: The state space encompassing all possible environmental configurations;
*A*: The action space containing all executable agent behaviors;
P: The state transition function $P\left(s^{\prime }\left|s,a\right.\right)\to \left[0,1\right]$, specifying the probability of transitioning to state s′∈S when executing action a∈A in state s∈S;
R: The reward function $R\left(s^{\prime }\left|s,a\right.\right)\to \mathbb{R}$, representing the expected immediate return obtained when transitioning to state s′ through action a in state s.

This formulation conforms to the Markov property, whereby subsequent states depend exclusively on the current state–action pair, thereby establishing the theoretical framework for sequential decision-making in reinforcement learning. The fundamental mechanism of MDP operates through an agent selecting actions from the action space according to a state transition function, subsequently applying these actions to the environment. This interaction induces state transitions while generating immediate reward signals. The primary objective of reinforcement learning in the SARL context is to enable the agent to derive an optimal policy that maximizes the expected long-term return through iterative environmental interactions.

### 2.2. Multi-Agent Game Modeling

Viewed through the lens of game-theoretic analysis, multi-agent interactive decision-making frameworks can be formally categorized into two principal classes: stochastic games (SGs) and extensive-form games (EFGs).

#### 2.2.1. Stochastic Games

The extension of MDP to multi-agent reinforcement learning is formally characterized as a stochastic game, which synthesizes the temporal dynamics of MDP with the strategic interdependence of normal-form games. Mathematically, an *n*-agent stochastic game can be represented by the tuple $\langle N,S,{\left\{A_{i}\right\}}_{i=1}^{N},P,{\left\{r_{i}\right\}}_{i=1}^{N},\gamma \rangle$ [12]:

*S*: The set of state spaces;
*N*: Cardinality of autonomous agents, $N=\left\{1,2,\ldots ,n\right\}$;
$A_{i}$: The action space of agent i, with joint action space $A=A_{1}\times A_{2}\times \ldots \times A_{N}$;
P: The state transfer probability function P:S×A×S→[0,1];
$r_{i}$: The reward function $r_{i}:S\times A\to R$;
γ: Temporal discount factor $\gamma \in \left[0,1\right)$.

**Figure 3:**
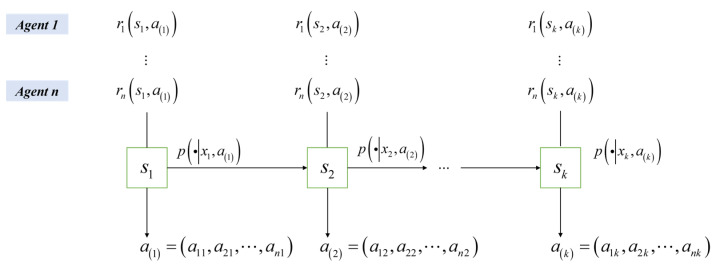
Joint action space optimization and state transfer mechanism in multi-agent stochastic games.

Figure 3 explains the dynamic interaction mechanism of the multi-agent stochastic game, in which agent i executes action $a_{i}$ in state $s_{i}$, which triggers the environment state transfer through the joint action space $a=\left(a_{1},a_{2},\ldots ,a_{n}\right)$ and at the same time triggers the individual rewards, which provides a structured framework for the subsequent analysis of the optimization of Nash equilibrium strategies.

Within the stochastic game framework, agents in a multi-agent system (MAS) simultaneously execute decision selections, whose joint action profile concurrently governs both environmental state transition dynamics and collective reward distribution mechanisms [13]. Each agent operates with an individualized reward architecture, with the fundamental objective converging on deriving an equilibrium policy configuration $\pi_{i}$ that optimizes agents’ discounted long-term returns under environmental constraints [14].

Stochastic game frameworks are primarily classified along the spectrum of agent interaction objectives into three archetypal formulations: collaborative team-theoretic models for fully cooperative tasks, adversarial zero-sum configurations for pure competition, and mixed-motive general-sum structures for hybrid scenarios. Cooperative team games predominantly apply to multi-agent coordination challenges such as UAV swarm formation control and connected vehicle platooning optimization. Competitive paradigms manifest in two distinct forms—strictly oppositional zero-sum interactions exemplified by combinatorial game theory applications (e.g., Go strategy optimization) and nuanced general-sum engagements requiring balanced cooperation–competition tradeoffs. The equilibrium computation mechanisms across these game-theoretic categories manifest fundamental divergences in their algorithmic implementations, operational contexts, and convergence guarantees, dictated by their respective incentive structures and payoff distributions.

As delineated in Table 1, contemporary algorithmic approaches for stochastic game equilibrium learning necessitate fundamental tripartite tradeoffs among communication efficiency, computational tractability, and environmental adaptability. In cooperative settings, Team-Q-learning achieves global optimization via centralized value decomposition yet incurs prohibitive communication overhead that impedes scalability in large-scale multi-agent deployments. Conversely, Distributed-Q-learning employs decentralized independent learners to minimize coordination costs but necessitates sophisticated credit assignment mechanisms to mitigate the risk of relative overgeneralization. For competitive paradigms, Minimax-Q demonstrates provable robustness in zero-sum interactions through worst-case optimization, though its dependence on opponent strategy transparency restricts applicability in imperfect information scenarios. Nash-Q extends equilibrium computation to general-sum games via coupled strategy updates yet suffers from combinatorial complexity explosions in high-dimensional action spaces. Hybrid scenarios witness Friend-or-Foe-Q’s role-based policy factorization enabling efficient computation in fixed-interaction patterns (e.g., robotic soccer formations), whereas Win or Learn Fast algorithms enhance adaptability through dynamic role allocation (e.g., StarCraft II multi-task coordination), albeit constrained by data inefficiency and overfitting vulnerabilities in sparse-reward environments.

While significant advances have been achieved in specialized domains, three persistent limitations impede broader applicability: (1) environmental dynamics adaptation deficiencies in non-stationary settings, (2) scalability bottlenecks in ultra-scale agent populations, and (3) coordination challenges across heterogeneous agent capabilities. Emerging research frontiers propose synergistic integration of hierarchical reinforcement learning architectures, meta-game-theoretic analysis, and graph-structured communication protocols to enhance real-time decision quality and fault tolerance in open-world multi-agent systems.

#### 2.2.2. Extensive-Form Games

**Figure 4:**
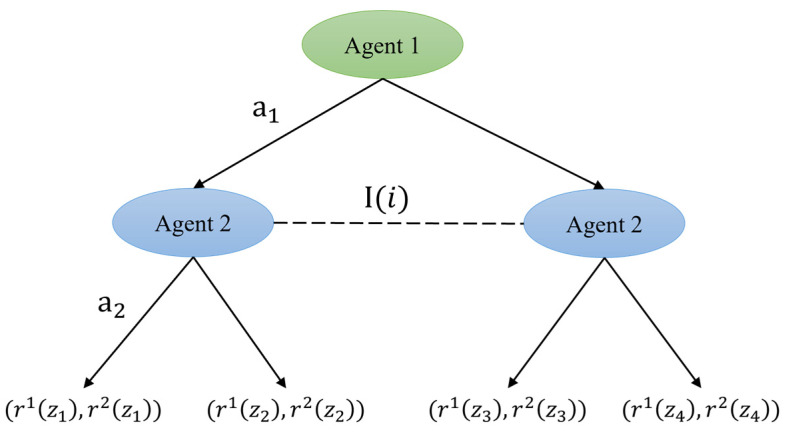
Multi-stage decision tree modeling under incomplete information in extended games.

Extensive-form games (EFGs) model sequential decision-making processes where agents engage in stage-dependent strategic interactions, formally represented through game tree formalism. As depicted in Figure 4, the multi-stage sequential interaction topology is mathematically characterized by tuples $\langle N,H,P,I,u,A\rangle$, where N denotes agent set and H represents the game history.

The extended-game framework consists of nodes and edges. Intermediate vertices (non-terminal nodes) correspond to decision points uniquely assigned to individual agents, with each intermediate vertex being exclusively controlled by a single decision-making entity. Terminal vertices (leaf nodes) encapsulate game outcomes, annotated with the respective utility values allocated to each participating agent. The directed arcs that interconnect these vertices represent the available strategic options at each decision juncture.

The extended game can be defined as $\Gamma =\langle N,H,P,{\left\{I_{i}\right\}}_{i\in N},{\left\{u_{i}\right\}}_{i\in N},A\rangle$.

N: The set of agent, denoting the cardinality of strategic decision-makers in the game.

H: The set of history, constituting all finite sequences from the root node to current positions.

P: The player function, mapping non-terminal histories to active agents or stochastic mechanisms.

$I_{i}$: An information set, which is a collection of decision points of an agent, is a single node in a complete information game, and a multi-node information set in an incomplete information game.

$u_{i}$: Defining the rewards of agent i in termination history.

A: Optional set of actions on each node.

**Figure 5:**
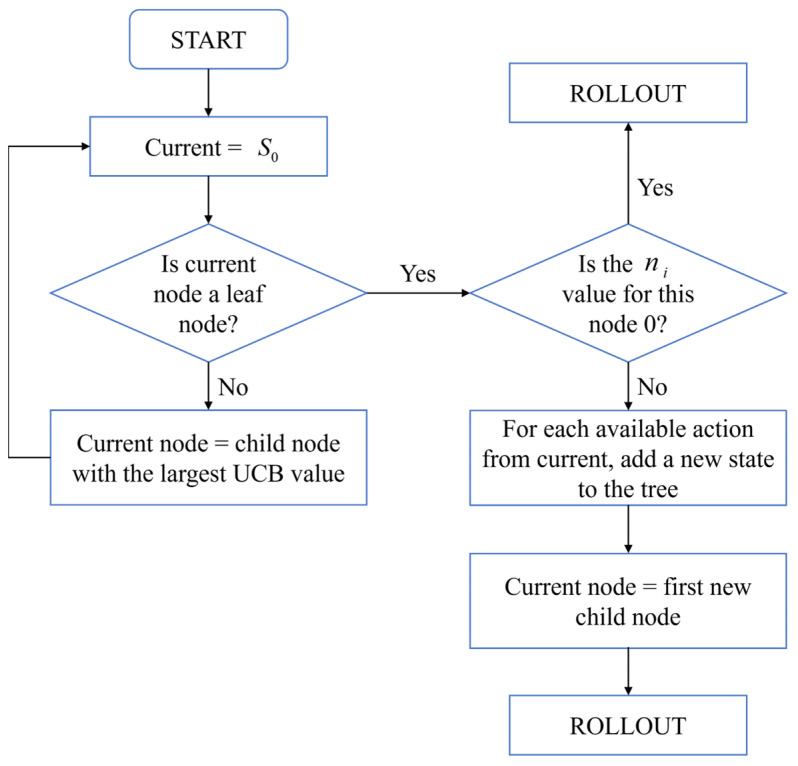
Monte Carlo Tree Search algorithm flow and equilibrium approximation.

Figure 5 depicts the dynamic search mechanism of the extended-game tree based on the upper confidence bound (UCB). The EFG framework encompasses two canonical representational schemes:

(a) The normal form representation, specifically designed for modeling simultaneous-move decision processes, serves as the standard paradigm for strategic interactions with parallel action selection [23].
(b) The sequence form representation, engineered to capture multi-stage behavioral strategies, provides an optimal mathematical framework for sequential decision-making scenarios involving intertemporal strategy commitments [24].

## 3. MARL Solution Method

### 3.1. Value-Based RL

#### 3.1.1. Bellman Equation and Nash Equilibrium

In reinforcement learning, the value function serves as a quantitative measure for evaluating the expected return of executing a specific action in a given state under a policy, encompassing both the state value function and the state–action value function. The objective of reinforcement learning is to maximize long-term rewards, where the cumulative reward for an agent starting from step *t* until termination at step *T* can be defined as

(1) $$G_{t}=R_{t+1}+R_{t+2}+\ldots +R_{T}$$

To address the issue of cumulative rewards tending towards infinity, value function-based methods introduce the discount factor γ, which signifies that the impact of earlier rewards diminishes over time. At this point, the cumulative reward can be expressed as

(2) $$G_{t}=R_{t+1}+\gamma R_{t+2}+\gamma^{2}R_{t+3}\ldots =\sum_{k=0}^{\infty }\gamma^{k}R_{t+k+1}$$

However, in practical applications, since subsequent actions and outcomes are not a priori known, implementing summation-based approaches proves challenging. This necessitates the adoption of “value” as a substitute for cumulative rewards. Reinforcement learning methods based on MDP modeling categorize value functions into two types: state value functions and state–action value functions.

The state value function $v_{\pi }(s_{t})$ characterizes the trajectory in reinforcement learning, representing the mathematical expectation of long-term rewards through sequences derived from the policy and state transition probabilities. It quantifies the expected total return obtained by following the policy starting from state s.

(3) $$V^{\pi }(s)=E_{a∼\pi (a|s)}\left[E_{s^{\prime }∼p(s^{\prime }|s,a)}\left[r(s,a,s^{\prime })+\gamma V^{\pi }(s^{\prime })\right]\right]$$

The state–action value function $Q^{\pi }(s,a)$ quantifies the expected total return obtained by taking action a in a given state s and subsequently following a specific policy.

(4) $$Q^{\pi }(s,a)=E_{s^{\prime }∼p(s^{\prime }|s,a)}\left[r(s,a,s^{\prime })+\gamma V^{\pi }(s^{\prime })\right]$$

In Markov Decision Processes, the Bellman equations for the state value function and the state–action value function are formulated, respectively, as

(5) $$V^{\pi }(s)=\sum_{a\in A}\pi (a|s)\sum_{s^{\prime }\in S}P(s^{\prime }|s,a)\left[R(s,a,s^{\prime })+\gamma V^{\pi }(s^{\prime })\right]$$

(6) $$Q^{\pi }(s,a)=\sum_{s^{\prime }\subset S}P(s^{\prime }|s,a)\left[R(s,a,s^{\prime })+\gamma \sum_{a^{\prime }\in A}\pi (a^{\prime }|s^{\prime })Q^{\pi }(s^{\prime },a^{\prime })\right]$$

The Bellman equations yield recursive formulations of the state value function and state–action value function employed in reinforcement learning. By assuming a known value function, these recursive relationships enable the iterative computation of the value for any state through other state value functions and state–action value functions.

In two-player zero-sum games, where the objectives of the two players are diametrically opposed, a strategy profile $\left(\pi_{*}^{1},\pi_{*}^{2}\right)$ that satisfies Equation (7) is defined as a Nash equilibrium [14].

(7) $$V^{1}(\pi_{*}^{1},\pi^{2})\geq V^{1}(\pi_{*}^{1},\pi_{*}^{2})\geq V^{1}(\pi^{1},\pi_{*}^{2})$$

Under the Bellman optimality principle, this Nash equilibrium transforms into a minimax equilibrium point, with the existence of a unique value function that is mathematically guaranteed [25].

(8) $$V_{*}(s)=\underset{\pi^{1}\in \Pi^{1}}{\max}\underset{\pi^{2}\in \Pi^{2}}{\min}V^{1}(s|\pi^{1},\pi^{2})$$

The Q-function-based Bellman minimax equation is formulated as

(9) $$\begin{array}{l} Q_{s}(s_{t},a_{t}^{1},a_{t}^{2})=R(s_{t},a_{t}^{1},a_{t}^{2})+\gamma \sum_{s_{t+1}}P(s_{t+1}|s_{t},a_{t}^{1},a_{t}^{2})\\ ⊙\underset{s_{t}^{2}}{\max}\underset{s_{t+1}^{1}}{\min}\sum_{s_{t+1}^{1}}\pi^{1}(a_{t+1}^{1}|s_{t+1})Q^{*}(s_{t+1},a_{t+1}^{1},a_{t+1}^{2}) \end{array}$$

In two-player zero-sum games, if the Q-function at the Nash equilibrium can be obtained, the dynamic game can be transformed into a normal-form game, thereby enabling the resolution of Nash equilibrium solutions via linear programming [26,27]. However, due to the high-dimensional nonlinear nature of the Bellman minimax equation, deriving direct analytical solutions is extremely challenging. In contrast, the dynamic programming (DP) approach operates independently of analytical solutions.

#### 3.1.2. Dynamic Programming Algorithm

The core tenet of dynamic programming algorithms lies in the iterative optimization of value functions or policies until the policy converges to optimality. By decomposing complex problems into subproblems and leveraging memorization techniques to cache intermediate results, DP effectively eliminates redundant computations and substantially enhances computational efficiency. This intrinsic property underpins its significant applicability in the domain of reinforcement learning.

**Figure 6:**
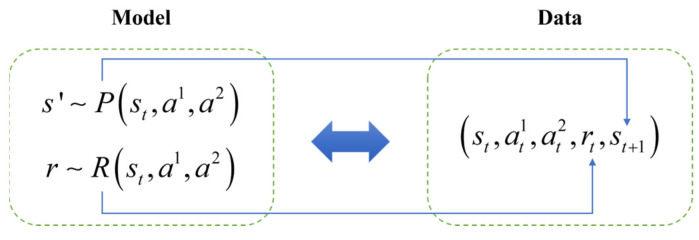
Dynamic planning value iteration illustration.

Dynamic programming algorithms approximate Nash equilibrium solutions through value iteration or policy iteration, as the flow of value iteration illustrated in Figure 6. However, DP relies on precise modeling of state transition probabilities and reward functions, exhibiting significant limitations in real-world scenarios where environmental dynamics are unknown or opponent strategies are time-varying. Additionally, dynamic programming methods are only applicable to small-scale game settings, as the curse of dimensionality in joint action space scales renders the algorithm non-scalable to large-scale multi-agent scenarios.

#### 3.1.3. Sample-Based Approach to RL

In contrast to dynamic programming, model-free Q-learning methods directly update Q-values through sampled interaction data without requiring prior knowledge of environmental models. Their inherent capabilities for online learning and continuous exploration enable effective adaptation to multi-agent game scenarios with dynamically evolving opponent strategies.

As a classical model-free algorithm in reinforcement learning, Q-learning employs temporal difference (TD) methods to achieve iterative Q-value updates [28]. However, traditional tabular Q-learning exhibits notable limitations: its discrete state–action space storage mechanism struggles to handle the curse of dimensionality arising from high-dimensional states, while its single-pass learning paradigm results in inefficient data utilization [29]. To overcome these constraints, the deep reinforcement learning framework Deep Q-Network (DQN) introduces three key innovations: (1) deployment of deep convolutional neural networks as Q-function approximators to resolve high-dimensional state space representation challenges; (2) integration of experience replay mechanisms that enable strategic data reuse while optimizing sample efficiency; and (3) utilization of target network architectures with delayed parameter synchronization to ensure algorithmic stability enhancement.

However, DQN suffers from value function overestimation due to systematic amplification of action value estimation errors caused by the maximization operation in its update rule. Double DQN (DDQN) addresses this by introducing a decoupled network architecture that separates action selection and Q-value evaluation networks, effectively suppressing estimation bias through dual-network alternating updates [30]. To enhance exploration efficiency, Noisy Net DQN replaces traditional ε-greedy exploration with learnable noise injection in neural network parameter space, enabling state-dependent autonomous exploration [31,32]. For value representation, Dueling DQN significantly improves environmental state value perception by decoupling Q-networks into dual-stream architectures comprising state value functions $V\left(s\right)$ and action advantage functions $A\left(a\right)$ [33,34]. Distributional DQN abandons scalar value estimation paradigms to model Q-value probability distributions, capturing latent uncertainty in environmental feedback [35]. Its enhanced variant, Implicit Quantile Networks (IQNs), characterizes environmental uncertainty through continuous quantile modeling [36]; and Quantile Regression DQN (QR-DQN) optimizes distribution estimation accuracy via a quantile regression loss [37]. Collectively, these innovations substantially strengthen algorithmic risk-aware decision-making capabilities in stochastic environments.

Value-based reinforcement learning methods necessitate the maintenance of high-dimensional Q-tables or sophisticated function approximators. When state and action spaces exhibit high dimensionality, storage and computational costs grow exponentially, resulting in the curse of dimensionality. While value function-based reinforcement learning is generally effective for discrete spaces, its requirement for maximizing Q-values in continuous action spaces leads to computationally inefficient operations, characterized by high computational complexity and poor convergence properties.

### 3.2. Policy-Based RL

Policy-based reinforcement learning achieves agent decision-making by directly optimizing the policy function. Its core principle involves parameterizing the policy as a differentiable function $\pi_{\theta }\left(a\left|s\right.\right)$ and updating parameters θ using gradient information derived from trajectory cumulative rewards. In contrast to value function-based approaches, policy gradient algorithms demonstrate significant advantages in handling high-dimensional continuous action spaces, operating within partially observable Markov Decision Process (POMDP) environments, and optimizing stochastic policies through direct parameterization of the decision-making strategy [38,39].

#### 3.2.1. Independent Reinforcement Learning

In multi-agent systems, independent reinforcement learning (IRL) adopts a decentralized architecture where each agent independently optimizes its policy within a single-agent framework [40]. In loosely coupled tasks, such as distributed robotic path planning, IRL achieves efficient scalability by circumventing the curse of dimensionality in joint policy spaces [41]. However, the asynchronous nature of agent policy $\pi_{\theta_{i}}$ updates induces time-varying characteristics in environmental dynamics $P\left(s\prime \left|s,a_{i}\right.\right)$, causing traditional policy gradient estimators $\nabla_{\theta_{i}}J\left(\theta_{i}\right)=E\left[\nabla \log\pi_{\theta_{i}}Q_{i}\right]$ to exhibit bias due to violations of the Markov assumption. Furthermore, IRL agents rely solely on Monte Carlo return estimates G from their individual trajectories for gradient computation, resulting in high variance and slow convergence.

In order to address these challenges, researchers have developed a series of methodological improvements. The Independent Proximal Policy Optimization (IPPO) algorithm inherits the trust region constraint mechanism from PPO, incorporating importance sampling and adaptive KL-divergence penalty terms for each agent. While retaining the independent learning framework, IPPO effectively mitigates gradient bias impacts by constraining policy update magnitudes, demonstrating enhanced stability in mildly collaborative tasks such as swarm robotic path planning.

To further reduce policy gradient estimation variance, the Independent Actor–Critic (IAC) method employs a divide-and-conquer architecture: each agent maintains dedicated actor and critic networks. The actor network updates parameters via the policy gradient theorem, while the critic network minimizes temporal difference errors for state value estimation. Although IAC’s architecture successfully lowers gradient variance, in dynamic multi-agent games, abrupt opponent strategy shifts and environmental non-stationarity may induce systematic bias (non-stationarity bias) in critic network value estimations.

In addressing the challenge of optimization in continuous action spaces, the Independent Deep Deterministic Policy Gradient (IDDPG) integrates the Deep Deterministic Policy Gradient (DDPG) with an independent learning paradigm [42]. By employing an experience replay mechanism and soft updates via target networks, the IDDPG algorithm significantly enhances policy learning efficiency and stability in continuous action spaces.

In communication-constrained scenarios where agents cannot perceive others’ strategies, the Fully Independent Multi-Agent Learning (FIMAL) framework treats all external agents as components of environmental dynamics. Operating solely on local observational data to directly optimize policy parameters, FIMAL reduces MAS dependencies on communication and joint information while exhibiting robust interference resistance. However, its complete independence precludes explicit coordination mechanisms, rendering agents prone to local equilibria in resource-competitive tasks and amplifying susceptibility to environmental non-stationarity during policy updates.

In summary, independent reinforcement learning methods offer unique advantages in scalability and privacy preservation through decentralized architectures, yet their performance remains constrained by environmental non-stationarity, exploration inefficiency, and coordination mechanism deficiencies. Future research should seek equilibrium between independent learning and centralized coordination—for instance, achieving policy adaptation via meta-learning or enhancing multi-agent collaboration through implicit communication mechanisms.

#### 3.2.2. Strategic Game and Coordination Mechanism Enhancement

To mitigate policy oscillation in independent learning and enhance multi-agent collaboration, researchers have developed game-theoretic policy optimization frameworks that improve system stability through strategic adversarial training and coordination mechanisms. Self-play, a prominent approach, drives policy evolution by dynamically generating adversarial strategies through competitive or cooperative interactions.

Self-play, first proposed by Gerald Tesauro, operates on the core principle of progressive “training curricula” where agents compete against historical versions of their own policies [43]. A canonical example is OpenAI’s Hide and Seek multi-agent environment, where two groups (hiders and seekers) undergo adversarial training via pure policy gradient methods. Hiders aim to construct shelters by manipulating obstacles to evade seekers, while seekers learn to breach barriers for successful captures. During initial training phases, both groups exhibit simplistic strategies (e.g., random movement). Through iterative training, agents progressively develop sophisticated historical policy versions—hiders learn to anchor obstacles, and seekers master using ramps as tools to circumvent blockades [44]. By continuously confronting historical strategies, the system autonomously generates difficulty-escalating training samples, ultimately emerging complex behaviors such as tool usage and dynamic coordination [45]. This process, entirely grounded in policy gradient optimization, validates self-play’s capacity for policy innovation without prior knowledge. However, in asymmetric games, self-play risks entrapment in “strategy cycling,” where agents oscillate periodically within constrained policy sets.

The challenge of strategy cycling in self-play has prompted methodological innovations. Heinrich et al. introduced Neural Fictitious Self-Play (NFSP), integrating fictitious play with deep Q-learning. Each agent maintains dual policy repositories: a best-response policy $\pi^{*}$ optimized via DQN for immediate rewards and an average policy $\pi^{-}$ updated through sliding-window historical action distribution tracking. In Texas Hold’em Poker experiments, NFSP reduced Nash gaps significantly by sampling opponent historical strategies from experience pools, eliminating explicit opponent modeling.

Building on this, the Prioritized Fictitious Self-Play (PFSP) algorithm enhances fictitious play by computing optimal responses to historical average policies for approximate Nash equilibrium solutions, incorporating prioritization mechanisms to refine training efficiency [46]. PFSP’s integration with deep learning yields Neural Fictitious Self-Play (NFSP), which achieves approximate Nash equilibria in imperfect-information games and scales to multi-agent partially observable settings [47].

#### 3.2.3. Evolutionary Reinforcement Learning Methods

Deep reinforcement learning (DRL) confronts intrinsic limitations when addressing complex decision-making tasks. The sparse-reward signals prevalent in partially observable environments hinder agents’ capacity to establish causal relationships between actions and long-term outcomes. Conventional DRL approaches relying on local gradient-based optimization frequently converge to suboptimal policies in high-dimensional continuous action spaces, exacerbated by exploration–exploitation imbalances that constrain sample efficiency. Furthermore, pronounced sensitivity to hyperparameter configurations often induces training instability and delayed convergence, particularly in stochastic environments with non-stationary dynamics [48].

Evolutionary algorithms (EAs) offer complementary strengths through population-driven optimization mechanisms. By maintaining diverse policy populations and applying genetic operators including crossover and mutation, EAs circumvent single-strategy myopia while fostering emergent behavioral diversity. Their gradient-free global optimization capabilities, enabled by parallelized population evaluation architectures, provide robust escape routes from local optima—a critical advantage in rugged fitness landscapes. Additionally, the inherent resilience of evolutionary processes to hyperparameter variations and initial policy distributions enhances algorithmic robustness in dynamically changing environments.

The symbiotic integration of reinforcement learning and evolutionary computation establishes a complementary optimization framework. Evolutionary mechanisms provide two critical enhancements: (1) global exploration through diversified policy initialization distributions and (2) accumulated exploration priors through generational selection processes. Conversely, gradient-based policy refinement in DRL enables rapid convergence to high-performance strategies, facilitated by temporal difference error minimization. This synergistic framework achieves adaptive exploration–exploitation tradeoff optimization through dual-phase coordination—evolutionary operators expand the strategic frontier while gradient methods exploit local optima.

Evolutionary Reinforcement Learning methodologies comprise four principal methodological paradigms: evolutionary parameter space exploration for neural architecture optimization, policy gradient hybridization through evolutionary direction vectors, population-based policy optimization with diversity preservation mechanisms, and evolution-guided deep reinforcement learning architectures employing fitness landscape analysis.

**Figure 7:**
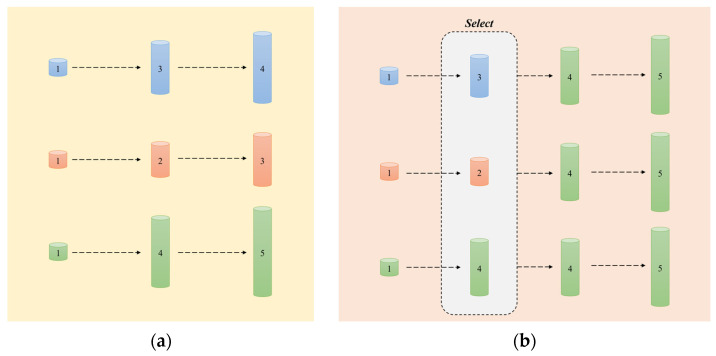
Comparative study of parameter optimization paradigms in reinforcement learning. (**a**) Decoupled parallel optimization and (**b**) population-based policy co-evolution. Different-colored bars represent distinct policy individuals in both subfigures.

The parameter distribution search paradigm conceptualizes policy optimization as probability density estimation, where evolutionary computation principles guide parametric evolution through reward-driven fitness landscapes. At its core, this methodology treats policy parameters as probability distributions, utilizing cumulative reward signals as fitness metrics to drive evolutionary optimization processes. The Parameter Exploration Policy Gradient (PEPG) approach exemplifies this framework by directly sampling policy parameters from Gaussian distributions and computing gradient updates through reward-weighted averaging of parameter perturbations [49]. Complementing this, Natural Evolution Strategies (NES) enhance optimization efficiency through natural gradient descent with adaptive covariance matrix adaptation, effectively navigating the Fisher information geometry of policy parameter spaces [50]. Simultaneously, the Cross-Entropy Method (CEM) implements iterative distribution refinement by retaining elite operational samples, incorporating strategic noise injection mechanisms to circumvent premature convergence while maintaining population diversity. As shown in Figure 7a, the traditional parallel optimization architecture achieves linear accumulation of strategy performance in a fixed hyperparameter space through independent gradient update paths, and its performance growth is limited by the isolated training mechanism without informative interactions; in contrast, the population interaction framework proposed in Figure 7b breaks through this limitation and drives the strategy performance by competitive elite operator selection through the SELECT module.

The evolutionary strategy paradigm has engendered significant methodological innovations in gradient-free policy optimization. Table 2 systematically classifies and compares key evolutionary reinforcement learning algorithms across three primary paradigms, highlighting representative methods and their defining characteristics. OpenAI’s foundational work in this domain materialized through OpenAI Evolution Strategy (OpenAI-ES), which replaces conventional gradient computation with population-based policy perturbation analysis. This approach evaluates policy performance through systematic parameter space exploration, effectively addressing gradient estimation challenges in high-dimensional optimization landscapes and establishing the theoretical framework for policy gradient approximation methods. Subsequent advancements introduced Novelty Search Evolution Strategy (NS-ES), integrating behavioral diversity metrics with evolutionary optimization [51]. By quantifying policy behavior uniqueness through trajectory divergence measurements, NS-ES drives exploration beyond reward maximization objectives. Further extending this paradigm, Novelty Search with Reward Evolution Strategy (NSR-ES) implements quality–diversity optimization through multi-objective Pareto front analysis. This hybrid architecture simultaneously optimizes reward maximization and behavioral novelty objectives via adaptive weight allocation across competing optimization criteria, achieving strategic balance between performance optimization and exploratory diversity preservation in complex decision spaces.

The strategy population search approach conceptualizes each autonomous policy as an independent evolutionary entity within a population, conducting comprehensive exploration across the strategic solution space guided by fitness-driven selection mechanisms. Population-Based Training (PBT) implements evolutionary-inspired optimization by dynamically adjusting policy hyperparameters and network weights through asynchronous population evaluation and performance-driven selection [52]. Its enhanced variant, the PB2 algorithm, incorporates probabilistic Population-Based Bayesian Optimization modeling to guide policy updates, improving theoretical convergence guarantees through Bayesian-optimized evolutionary transitions. The Deep Evolutionary Reinforcement Learning (DERL) framework pioneers co-evolutionary adaptation by synergistically optimizing neural control policies with morphological parameters, generating diverse agent embodiments capable of dynamically adjusting to environmental complexities through integrated evolutionary–developmental learning mechanisms.

Evolutionary-guided deep reinforcement learning methodologies effectively reconcile exploration efficiency with precision through synergistic integration mechanisms. The Evolutionary Reinforcement Learning (ERL) framework implements parallelized co-evolution of population-based search and gradient-based optimization, facilitating bidirectional knowledge transfer through shared experience replay buffers and policy network parameter synchronization. The Cross-Entropy Method Reinforcement Learning (CEM-RL) architecture enhances high-dimensional control stability by integrating subpopulation evaluation with gradient-based optimization, combining TD3’s actor–critic framework with evolutionary exploration heuristics. Building upon ERL, Proximal Distilled Evolutionary Reinforcement Learning (PDERL) introduces policy distillation techniques and proximal gradient constraints to mitigate policy network degradation caused by evolutionary operators’ abrupt parameter perturbations [53]. Advancing beyond conventional paradigms, Quality–Diversity Reinforcement Learning (QD-RL) implements a dual-objective optimization framework.

Reinforcement learning’s reliance on environment-sampled data introduces high correlation and dynamic distribution shifts, resulting in low data efficiency and susceptibility to local optima [48]. DeepMind’s population-based learning framework addresses this by synergizing evolutionary algorithms with RL [54]. Deploying identical RL algorithms across distributed processes or machines, it trains and maintains agent populations where individuals assimilate optimal experiences from peers, accelerating convergence while preserving diversity [55].

Ming Zhou et al. proposed MALib, a scalable and efficient population-based multi-agent reinforcement learning (PB-MARL) computational framework, to tackle the complex nested challenges of heterogeneous policy interaction sampling, training, and evaluation. By integrating dynamic heterogeneous task orchestration with decoupled parallel architecture design and cross-platform abstraction interfaces, MALib achieves efficient resource scheduling for multi-agent reinforcement learning tasks, enables synergistic optimization between training and sampling phases, and supports flexible deployment protocols, thereby significantly enhancing system throughput and scalability [56].

#### 3.2.4. Optimization in Continuous Action Spaces

While independent reinforcement learning and multi-agent game-theoretic coordination mechanisms have demonstrated significant policy optimization capabilities in discrete action spaces, extending reinforcement learning to continuous action spaces while maintaining efficient convergence remains a critical challenge for enhancing algorithmic generalization in complex scenarios such as physical control and sequential decision-making.

Existing research on strategic game reinforcement learning predominantly focuses on abstract problems in simulated environments, which often feature artificially defined rules, simplified state spaces, and an absence of real-world physical or dynamic constraints. In contrast, real-world systems typically involve high-dimensional nonlinear dynamics and intricate dynamic coupling effects, necessitating the integration of refined constraint modeling and adaptive optimization mechanisms into algorithmic design. Hierarchical reinforcement learning (HRL) frameworks address the coupling between control precision and decision flexibility by decomposing policies into low-level control (e.g., drone attitude optimization) and high-level game-theoretic strategies (e.g., tactical decision-making in MAS) [57].

To mitigate exploration inefficiency under sparse rewards, the Soft Actor–Critic (SAC) algorithm incorporates a policy entropy regularization term, compelling agents to maintain action diversity even during failures. This approach reduces sensitivity to initial policies and enhances exploration efficiency in sparse-reward scenarios. SAC has demonstrated substantial engineering value in applications ranging from solar spectrum separation and microgrid system optimization to vehicle energy management under complex operating conditions [58,59,60].

The Twin Delayed Deep Deterministic Policy Gradient (TD3) algorithm significantly improves stability and policy performance in continuous control tasks through three core mechanisms: dual Q-networks, delayed updates, and target policy smoothing. It has become a benchmark algorithm in robotics control and autonomous driving domains [61].

Policy gradient-based reinforcement learning methods prove adaptable to high-dimensional continuous action spaces and perform effectively in asymmetric games and partially observable scenarios. However, their joint optimization of policies and value functions incurs substantial computational overhead, and convergence guarantees in multi-agent settings remain theoretically underdeveloped.

### 3.3. Search-Based RL

#### 3.3.1. Monte Carlo Tree Search

Traditional reinforcement learning methods face two core challenges in dynamic game scenarios: first, pre-trained models based on value functions or policy functions often lack environmental generalization capabilities, struggling to adapt to game contexts beyond the training distribution; second, fixed policies cannot perform online optimization in response to environmental changes during execution. Monte Carlo Tree Search (MCTS) effectively resolves these limitations through its dynamic game tree construction and online planning mechanisms, with its core advantages embodied in two aspects:

1. Universality: The tree-search framework is universally applicable to any finite state space game problem.
2. Adaptability: Real-time updates of node statistics enable dynamic optimization of search paths.

**Figure 8:**
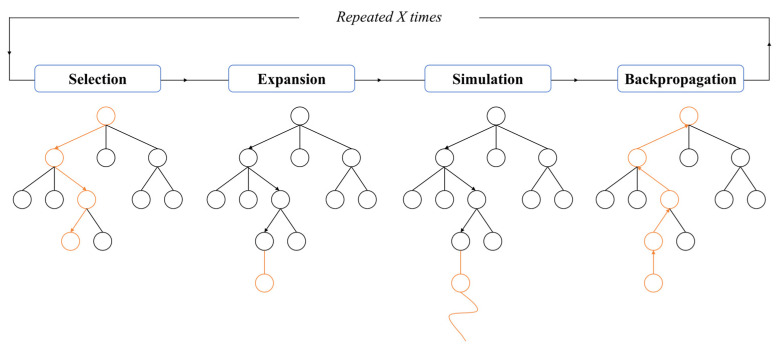
Monte Carlo Tree Search process.

As illustrated in Figure 8, the classical implementation of the Monte Carlo Tree Search (MCTS) algorithm involves an iterative four-phase process:

Step 1: Selection Phase. Beginning at the root node, the algorithm recursively selects optimal child nodes according to the upper confidence bound (UCB) criterion until reaching a leaf node. The UCB formula is defined as

(10) $$UCB=\frac{V_{i}}{n_{i}}+c\sqrt{\frac{\ln N}{n_{i}}}$$

where $V_{i}$ represents the node’s average value, c is a constant (typically set to two), N denotes the total number of explorations, and $n_{i}$ indicates the exploration count for the current node.

Step 2: Expansion Phase. When a leaf node does not correspond to a terminal state, child nodes are created based on the available action space, with one selected as the expanded node.

Step 3: Simulation Phase. A Monte Carlo random rollout is executed from the expanded node until reaching a terminal state, generating a reward signal.

Step 4: Backpropagation Phase. The simulation results are propagated backward along the search path, updating visit counts and value statistics for all traversed nodes.

In complex game scenarios, Monte Carlo Tree Search (MCTS) demonstrates significant advantages. Early research on Go problems typically combined expert systems with fuzzy matching methods to reduce search spaces, but practical effectiveness was constrained by computational resources and hardware capabilities of the time. With advancements in computing power, DeepMind’s AlphaGo [62] achieved superhuman decision-making in Go through synergistic integration of deep reinforcement learning and MCTS. This breakthrough marked the first instance where artificial intelligence surpassed professional human players in Go strategy by leveraging the collaborative mechanism between deep neural networks and tree search.

However, MCTS faces critical challenges in real-time gaming systems: the branching factor of game trees grows exponentially with decision dimensions, leading to prohibitive computational complexity, while the sequential selection–expansion–backpropagation workflow struggles to exploit parallel computing architectures. To address these limitations, researchers have developed enhanced algorithms such as Distributed MCTS (DMCTS), hierarchical pruning, and dynamic UCB adaptation strategies. DMCTS improves search efficiency in complex tasks by implementing parallelization across tree structures, root nodes, and simulation processes, effectively mitigating the inefficiencies of single-threaded computation [63]. Hierarchical pruning dynamically eliminates low-probability branches at different search depths, resolving computational resource exhaustion and real-time decision-making difficulties caused by excessively deep search trees and node explosions [64]. The dynamic UCB adaptation strategy overcomes the limitation of fixed exploration weights in standard MCTS by adjusting exploration coefficients based on game state evolution [65].

Further advancing this paradigm, David Silver et al. proposed the Asynchronous Policy and Value MCTS (APV-MCTS) algorithm, which integrates deep neural networks with MCTS. During the expansion phase, APV-MCTS utilizes policy networks to provide action prior probabilities, reducing redundant branch exploration. In backpropagation, it combines value network evaluations with Monte Carlo simulation outcomes to decrease reliance on lengthy path simulations, thereby validating the generalization potential of “neural network + tree search” architectures [62].

#### 3.3.2. Rolling Horizon Evolution Algorithm

**Figure 9:**
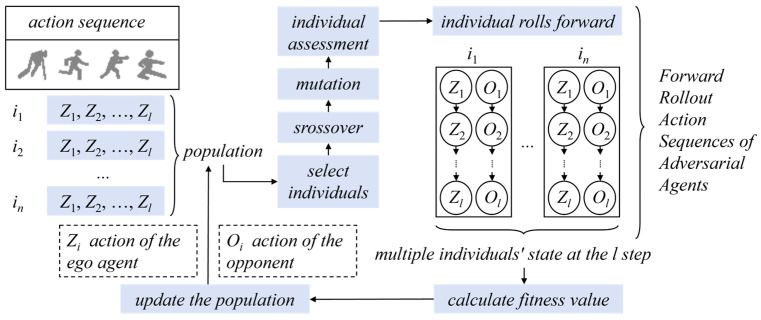
Deconstruction of rolling time–domain evolutionary algorithmic processes for real-time adversarial decision-making.

The Rolling Horizon Evolution Algorithm (RHEA) is an optimization methodology that integrates principles from evolutionary algorithms (EAs) and Rolling Horizon Control (RHC), primarily addressing sequential decision-making challenges in dynamic environments or real-time scenarios such as game AI, real-time control, and dynamic path planning [66]. As depicted in Figure 9, the Rolling Horizon Evolution Algorithm (RHEA) operates through two synergistic mechanisms: (1) a receding horizon window mechanism that achieves continuous environment state tracking through temporal model predictive control principles, and (2) population-based iterative optimization that conducts systematic exploration of measurement spaces via evolutionary computation paradigms. This architecture is specifically designed to address sequential decision-making challenges in non-stationary environments, demonstrating particular efficacy in interactive game AI decision-making, real-time robotic manipulation, and dynamic trajectory planning in autonomous navigation systems. Its core mechanism embeds evolutionary optimization within a rolling horizon framework, progressively approximating global optima through iterative window updates and population evolution.

Functioning as an environment-responsive intelligent decision-making system, RHEA establishes a state transition inference engine via forward models and employs population-based evolutionary mechanisms for prospective strategy simulation. The algorithm’s essence lies in constructing a closed-loop optimization system incorporating reward prediction, where adaptive decision generation is achieved through dynamic evaluation of candidate strategies’ expected returns.

However, RHEA’s modeling process unidirectionally focuses on agent–environment interactions while neglecting concurrent modeling of opponents’ decision impacts, resulting in environmental dynamic cognitive bias during forward simulations. This limitation manifests particularly in adversarial strategy generation for real-time two-player zero-sum games. To bridge this gap, Zhentao Tang et al. proposed the Rolling Horizon Evolution Algorithm with Opponent Model Learning (RHEAOM) [67], which integrates opponent modeling techniques to address traditional RHEA’s deficiencies in opponent behavior prediction and real-time adversarial adaptation, significantly enhancing decision adaptability and competitiveness in adversarial gaming AI.

While search-based reinforcement learning methods excel in decision optimization, they face persistent challenges in computational efficiency and real-time responsiveness. Additionally, their reliance on precise environmental simulators for rollouts often yields black-box decision policies lacking interpretability. Future research should prioritize three directions: deep integration of efficient opponent models with search algorithms, design of lightweight real-time decision frameworks, and enhancement of cross-task generalization capabilities—critical advancements for improving robustness and flexibility in complex environments.

## 4. Applications and Challenges of MARL

Dynamic Complexity and Heterogeneous Coordination Challenges in Complex Environments. Existing MARL algorithms predominantly assume static or short-term dynamic environments, whereas real-world applications require agents to maintain robustness in long-term time-varying conditions. In intelligent traffic signal control, traffic flow exhibits periodic fluctuations influenced by seasonal and meteorological factors [68]. Traditional Q-learning strategies suffer from control lag due to their neglect of long-term trends, while parameter-sharing methods like MAPPO fail under divergent policy space dimensionalities [69]. Although QMIX based on monotonicity constraints enables team value decomposition, it still exhibits substantial deviations in modeling nonlinear heterogeneous relationships. These issues are further exacerbated in UAV swarm cooperative search-and-rescue missions, where stochastic perturbations from sensor noise and communication delays force agents to adopt suboptimal strategies, invalidating classical game equilibrium predictions. More critically, continuous policy updates induce dynamic shifts in environmental state transition probabilities, undermining the foundational Markov assumption. While role discovery mechanisms such as Role-based Observation-Driven Exploration (RODE) leverage attention mechanisms to allocate agent responsibilities, their adaptability remains constrained by manually defined role priors, struggling to accommodate emergent state transitions in open environments.

Partial Observability and Adversarial Reasoning Bottlenecks. In imperfect-information games like Texas Hold’em, MARL must surpass the limits of information inference: agents must construct Bayesian belief state models of opponents through local observations to infer real-time hand probability distributions and strategy types. Though Deep Counterfactual Regret Minimization (Deep CFR) approximates counterfactual regret values via neural networks, exponential growth in information set sizes drastically escalates computational complexity [70]. Urban traffic scheduling scenarios reveal typical challenges of Partially Observable Markov Decision Processes (POMDPs), where mapping local observations to global road network states involves non-convex optimization, and multimodal belief distributions trigger convergence oscillations. Lin Li and other scholars proposed a multimodal driving intention POMDP model to model behavioral decision-making and motion planning in a unified way and developed a time-series recursive policy gradient algorithm (RDPG) to optimize driving strategies and trajectory generation simultaneously in partially observable environments [71]. Moreover, in supply chain coopetition games, suppliers may deploy strategic deception through false capacity signals, yet traditional sequential equilibrium frameworks fail to detect such “irrational equilibria,” leading to decision biases.

Computational Dimensionality Crisis and Real-Time Constraints. High-dimensional state spaces expose critical computational bottlenecks in multi-agent reinforcement learning. A representative example arises in Atari gaming environments, where pixel-level observations escalate state space dimensionality to magnitudes exceeding $10^{6}$ [31]. Hierarchical reinforcement learning frameworks like Option-Critic alleviate dimensionality pressures through meta-policy orchestration of subtasks; however, their generalization capabilities remain constrained by prior knowledge dependencies for subtask boundary initialization [72]. In real-time adversarial scenarios such as Dota 2, millisecond-level response requirements impose stringent computational demands. OpenAI Five achieved an average decision latency of 217 ms by integrating Proximal Policy Optimization (PPO) with distributed training frameworks, outperforming human visual reaction times of approximately 250 ms [73]. SEED RL further enhances real-time performance through centralized inference architectures, supporting millions of environmental interactions per second [74]. For lightweight model architectures, MobileNetV2 reduces training time to 2.1 h in agricultural remote sensing change detection by replacing ResNet50 backbones with inverted residual structures—a 0.41 h reduction compared to baseline models—though challenges persist in complex edge segmentation accuracy and hardware acceleration dependencies [75], requiring co-processing with specialized hardware like TPUs. The optimization paradox of hybrid discrete-continuous action spaces has been extensively analyzed in Soft Actor–Critic (SAC) extensions. Haarnoja et al. demonstrated in the foundational SAC work that discrete actions necessitate Gumbel–Softmax reparameterization [76], while “Hybrid SAC” experiments revealed increased policy gradient variance during joint discrete-continuous action optimization [77].

Theory–Practice Discrepancy. A significant chasm persists between theoretical MARL models and real-world requirements. In dynamic spectrum auction scenarios, where distributed base stations adjust transmission power based on local observations, asynchronous policy updates may trigger oscillatory global channel interference cycles, exposing the incompatibility of perfect rationality assumptions with dynamic environments. Halpern’s equilibrium logic framework posits that perfect equilibria require infinite recursive reasoning [78], yet prediction errors in renewable energy output systematically violate sequential equilibrium convergence conditions. Cross-border logistics multi-objective conflicts exemplify non-guaranteed Nash equilibrium existence, where competing goals such as cost minimization and delivery time optimization amplify policy uncertainty. While meta-game reasoning methods generate strategic populations to counter opponent diversity, their offline training paradigms struggle to meet real-time adversarial demands. Conversely, deep reinforcement learning-based online opponent modeling techniques face computational resource constraints, creating a methodological dilemma between responsiveness and scalability.

## 5. Conclusions and Future Directions

Multi-agent reinforcement learning (MARL) optimizes policies through trial-and-error learning yet faces challenges including environmental non-stationarity, policy interdependencies, and credit assignment difficulties arising from concurrent agent learning. Integrating MARL with game theory enables modeling strategic interactions through equilibrium analysis to predict steady-state rational outcomes, while mechanism design aligns individual objectives with collective goals. This synergy leverages MARL’s autonomous adaptability for dynamic scenarios and game theory’s strategic rigor to ensure system stability and efficiency—particularly in mixed competition–cooperation settings, resource conflicts, or fairness-driven collaborations (e.g., autonomous driving, economic games)—yielding more robust and interpretable multi-agent coordination and competition. However, current research struggles with dynamic environment adaptation, asymmetric information processing efficacy, and computational scalability. Future breakthroughs may emerge from three dimensions.

1. Dynamic Environment Adaptation Mechanisms

Develop time-varying adaptive meta-game frameworks using online meta-learning to capture environmental dynamics and enable rapid policy transfer. Integrate Bayesian inference networks to construct probabilistic models of dynamic parameters. Advance heterogeneous agent co-evolution theory with hierarchical attention mechanisms for dynamic role allocation. Employ graph neural networks (GNNs) to model evolving inter-agent topological relationships, addressing group strategy drift in open-world scenarios.

2. Imperfect-Information Game Reasoning Paradigms

Design hybrid belief state estimators combining deep variational inference with symbolic logic rules, reducing reliance on Bayesian priors. Strengthen integration of counterfactual reasoning and adversarial example generation via policy space perturbation and information set augmentation, enhancing strategy generalization in asymmetric games.

3. Ultra-Large-Scale Game Computing Architecture

Innovate tensor decomposition-based joint policy representation methods, employing low-rank approximations to compress strategy spaces for high-dimensional Nash equilibrium solutions. Build edge-cloud collaborative frameworks with dynamic computational load balancing via policy distillation and conditional computation. Concurrently, develop neural architecture search-driven lightweight models to achieve synergistic optimization of parameter compression and inference efficiency in UAV swarm gaming scenarios.

## Figures and Tables

**Table 1:** Comparison of stochastic game equilibrium learning methods and performance.

Game Type	Methodology	Applicable Scenario and Performance
Cooperative Game	Team-Q [15]	Suitable for fully collaborative scenarios requiring global state sharing; high communication costs; poor scalability in large-scale systems.
	Distributed-Q [16]	Distributed collaboration with partial observations; may fall into local optima, requiring additional coordination mechanisms.
	Joint Action Learner [17]	Distributed collaboration with partial observations; may fall into local optima, requiring additional coordination mechanisms.
	Frequency Maximum Q [18]	Effective in dynamic environments via frequency–domain exploration balancing; exhibits slow convergence speed.
Competitive Game	Minimax-Q [19]	Zero-sum games with strong robustness; requires known opponent strategies; and poorly adapts to imperfect-information games.
Mixed Cooperative–Competitive Game	Nash-Q [20]	Multi-agent competitive scenarios, supports Nash equilibrium solutions; low computational efficiency in high-dimensional policy spaces.
	Friend-or-Foe-Q [21]	Multi-team adversarial environments; high strategic flexibility; relies on accurate prior friend/foe knowledge; prone to policy oscillation in heterogeneous strategy spaces.
	Win or Learn Fast [22]	Non-stationary competitive environments; short-term strategies converge quickly, but may overfit local game models in the long run.

**Table 2:** Classification and comparison of Evolutionary Reinforcement Learning algorithms.

Categorization	Representative Algorithm	Characteristic
Parameter Distribution Search	PEPG	Parameter perturbation combined with gradient update
	NES	Natural gradient and covariance adaptation
	CEM	Elite sample-driven distribution update
Policy Gradient Approximation	OpenAI-ES	Evolution strategy for gradient-free optimization
	NS-ES	Novelty-driven behavior exploration
	NSR-ES	Quality–diversity Pareto optimization
Policy Population Search	PBT	Dynamic hyperparameter adjustment
	PB2	Bayesian optimization-enhanced PBT
	DERL	Morphological evolution for diverse agent generation
Evolution-Guided DRL	ERL	Parallel evolution and DRL with policy sharing
	CEM-RL	CEM-TD3 hybrid with population evaluation
	PDERL	Backpropagation-constrained mutation
	QD-RL	Archive policy Pareto selection

## Data Availability

The raw data supporting the conclusions of this article will be made available by the authors on request.
